# Predicting ICU mortality in heart failure patients based on blood tests and vital signs

**DOI:** 10.3389/fcvm.2025.1590367

**Published:** 2025-06-25

**Authors:** Yeao Wang, Jianke Rong, Zhili Wei, Xiaoyu Bai, YunDan Deng

**Affiliations:** ^1^ The First Clinical Medical College, Lanzhou University, Lanzhou, Gansu, China; ^2^ The Cardiovascular Surgery, The First Hospital of Lanzhou University, Lanzhou, Gansu, China

**Keywords:** machine learning, heart failure, NN, MLP, XGBoost, eICU, MIMIC

## Abstract

**Background:**

Currently, heart failure has become one of the major complications in the advanced stages of various cardiovascular diseases. Numerous predictive models have been developed to estimate the mortality rate of heart failure patients; however, these models often require the measurement of multiple indicators and the inclusion of various scoring systems. Critically ill patients are often unsuitable for extensive diagnostic tests, and many primary care hospitals lack comprehensive diagnostic equipment. In contrast, blood tests are not only simpler but also reflect the overall health status of the body. Therefore, using simpler methods to predict mortality in intensive care unit (ICU) patients has become the focus of this study.

**Method:**

A total of 5,383 cases from the eICU database were utilized for model development, while 530 cases from the MIMIC-IV database were employed for external testing. The patients were primarily diagnosed with heart failure, and the data included demographic information, blood oxygen saturation, white blood cells, red blood cells, platelets, hemoglobin, electrolytes, lactate, glucose, and other biochemical and physiological indicators collected during the ICU stay. Enhance the accuracy of data analysis and improve the universality of the model, all data underwent rigorous preprocessing prior to training, combined with data standardization. We utilized a variety of machine learning algorithms for modeling purposes, including Logistic Regression (LR), Support Vector Machine (SVM), Decision Trees, Random Forests, Gradient Boosting Machines (GBM), XGBoost, and Neural Networks. The performance of the model was assessed through cross-validation and evaluated using the F1-score.

**Conclusion:**

Through feature selection, 15 key variables were ultimately identified. Among the nine machine learning models evaluated, the Multilayer Perceptron (MLP) demonstrated the best overall performance. In predicting mortality (i.e., the deceased population), the MLP achieved an F1 score of 0.54, a recall of 0.71, and a precision of 0.44. The relatively high F1 score of the MLP highlights its potential clinical application value.

## Introduction

Heart failure (HF) is a significant cardiovascular condition with a high prevalence and mortality rate worldwide (1). With an aging population and the increasing prevalencerefere of heart disease, the number of heart failure patients continues to rise, positioning HF as a significant global public health concern (2, 3). Approximately 64 million individuals worldwide are currently affected by heart failure (HF), and this figure is anticipated to rise in the coming decades, particularly in low- and middle-income countries (3–5). HF imposes significant health and economic burdens on patients and their families, while also presenting a considerable challenge to healthcare systems. In the Intensive Care Unit (ICU), heart failure patients often present with more complex and critical conditions. HF is often associated with a range of comorbidities, including hypertension, coronary artery disease, chronic obstructive pulmonary disease, and chronic kidney disease (6, 7). As a result, ICU physicians must manage a vast array of clinical data, such as patient medical history, laboratory test results, imaging data, medication use, and real-time vital sign monitoring. The diversity and complexity of this data make it challenging for physicians to rapidly extract key clinical information, increasing the risk of misdiagnosis and treatment delays (8). To address this challenge, the medical field has increasingly adopted artificial intelligence (AI) and big data technologies to assist ICU physicians (9–11).

## Materials and methods

### Data sources and outcomes

This study presents a retrospective cohort analysis utilizing data extracted from the MIMIC-IV 3.1 and eICU databases. MIMIC-IV 3.1 is derived from data collected from patients in the Emergency Department and Intensive Care Unit (ICU) at Beth Israel Deaconess Medical Center in Boston (12). This hospital is equipped with extensive clinical resources and a comprehensive electronic medical record system, offering a large volume of real and detailed patient data. The database includes demographic data (age, gender, race, admission type, and admission source) as well as clinical data (heart rate, blood pressure, body temperature, respiratory rate) and laboratory results (complete blood count, blood biochemistry, coagulation function). It also records medical orders (medications, surgical procedures, diagnostic tests) and nursing records (fluid intake and output, such as infusion volume, urine output, and drainage volume). The eICU (2.0) database is a widely used multicenter database for critical care research, collecting clinical data from more than 200 ICUs across the United States (13). It encompasses hospitals of various regions, sizes, and types, including both teaching and community hospitals. This multicenter data source significantly enhances the diversity and representativeness of the data, making research conclusions more broadly applicable (14, 15). This study primarily aims to predict in-ICU mortality in HF patients.

### Patient research and definition

Patients in the MIMIC-IV and eICU database were screened based on the following criteria: aged over 18 years, admitted to the ICU for the first time, and diagnosed with heart failure as the primary condition. Since the mortality rate of heart failure varies significantly based on comorbidities, patients with other acute conditions were excluded from this study (16).

### Data collection, variable extraction, and missing value handling

Data from the two databases were extracted using SQL for 61 independent variables, including age, gender, minimum, maximum, and mean values of various biomarkers and physiological indicators (e.g., SpO_2_, wbc, rbc, platelet count, hemoglobin, sodium, lactate, glucose, creatinine, albumin, ALT, total bilirubin, troponin I, BNP, heart rate, respiratory rate, blood pressure, temperature, sapsii and sofa). The response variable was defined as in-hospital mortality, as it provides a direct measure of the patient's vital status during hospitalization.

The data processing strategy was as follows: Columns with more than 30% missing values were excluded from the analysis, while those with less than 30% missing data were imputed using multiple imputation (17–20).

**Figure 1:**
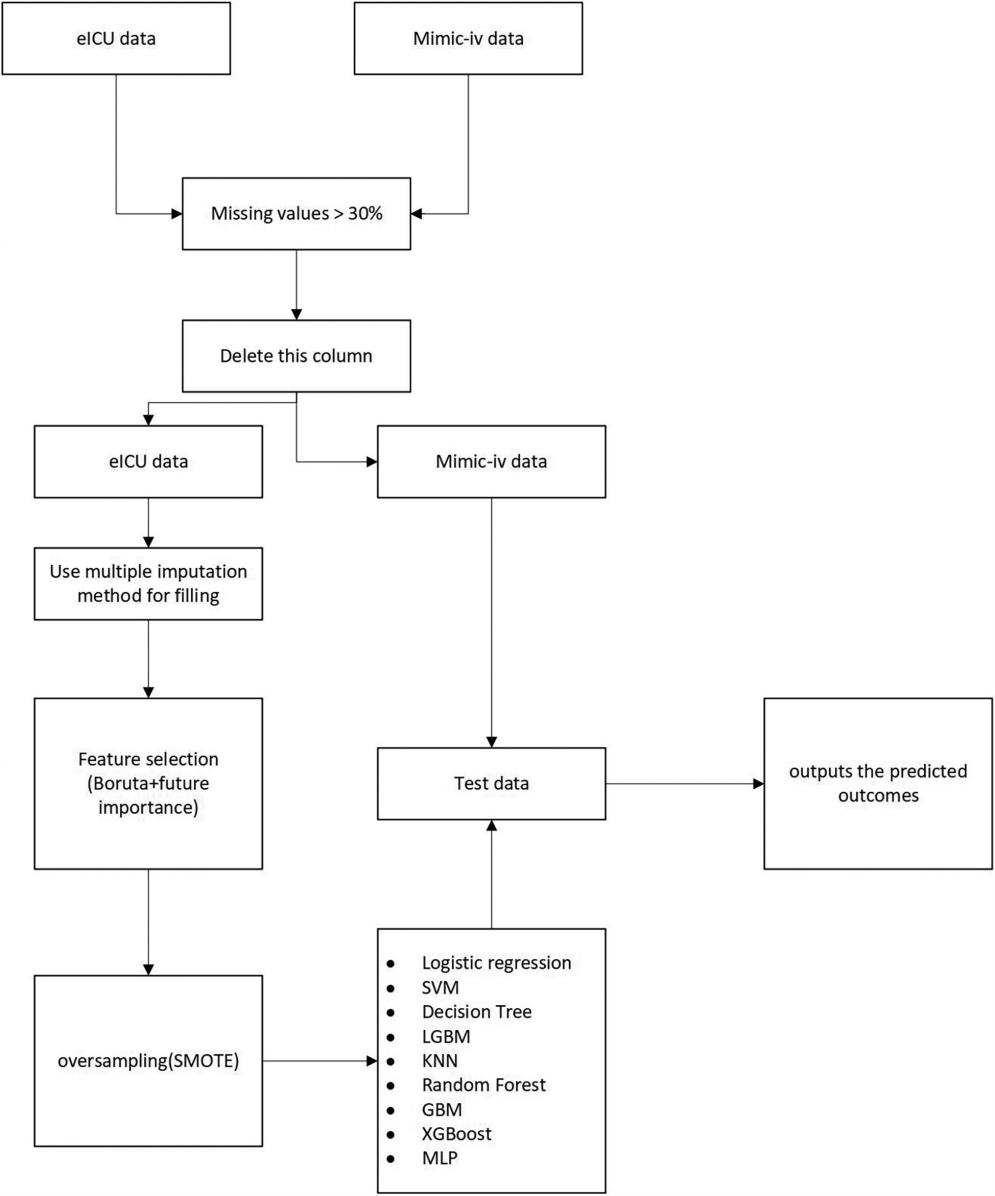
Flowchart of this research.

The flowchart is presented in Figure 1.

### Machine learning model construction

Regression models (LR), Support Vector Machines (SVM), Decision Trees (DT), LightGBM (LGBM), K-Nearest Neighbors (KNN), Random Forest (RF), Gradient Boosting Machines (GBM), eXtreme Gradient Boosting (XGBoost) and Multilayer Perceptron (MLP) were employed for prediction. Random search combined with 5-fold cross-validation was employed for model development. Random search optimizes performance and accelerates the process, but it may become trapped in local optima. In contrast, grid search requires enumerating all possible combinations to obtain the theoretically optimal solution, which is computationally expensive. Therefore, the advantages of random search generally outweigh its disadvantages (21–23).

### Model evaluation

The final model was evaluated using various metrics derived from the confusion matrix, including accuracy, F1-score, recall, the area under the receiver operating characteristic (ROC) curve (AUC) (24, 25). Given that model accuracy ranges from 80% to 90%, tuning efforts primarily focused on the F1-score, with an emphasis on optimizing predictive performance for the minority class. The F1-score was also used as the scoring criterion during cross-validation.

### Statistical analysis

As the model was developed using the eICU dataset, patients were categorized into two groups based on their survival status during ICU hospitalization. Subsequently, the median, first quartile (Q1), and third quartile (Q3) were calculated for each variable. Categorical and continuous variables were analyzed using the chi-square test and the Wilcoxon rank-sum test, respectively. To reduce the risk of false positives due to multiple comparisons, the Benjamini-Hochberg (BH) correction method was applied (26). Model performance was evaluated using the F1-score during model development with the training dataset.

## Result

### Baseline characteristics and features

As shown in the Table 1, a total of 5,383 patients with a primary diagnosis of heart failure were identified from the eICU database, among whom 286 died and 5,097 survived. The figure summarizes the baseline characteristics, vital signs, laboratory parameters, and SAPS II and SOFA scores of the survival and non-survival groups. Among non-survivors, variables such as SAPS II, SOFA score, minimum SpO_2_, mean SpO_2_, minimum white blood cell (WBC) count, maximum WBC, mean WBC, minimum red blood cell (RBC) count, mean RBC, minimum platelet count, minimum hemoglobin (HGB), mean HGB, maximum anion gap, mean anion gap, minimum sodium, maximum potassium, mean potassium, minimum bicarbonate, maximum bicarbonate, mean bicarbonate, minimum calcium, mean calcium, minimum blood urea nitrogen (BUN), maximum BUN, mean BUN, maximum creatinine, mean creatinine, minimum albumin, maximum albumin, mean albumin, minimum alanine aminotransferase (ALT/SGPT), maximum ALT, mean ALT, minimum total bilirubin, maximum total bilirubin, mean total bilirubin, minimum heart rate (HR), maximum HR, mean HR, minimum respiratory rate (RR), maximum RR, mean RR, minimum systolic blood pressure (SBP), maximum SBP, mean SBP, minimum diastolic blood pressure (DBP), mean DBP, minimum temperature, and maximum temperature showed statistically significant differences compared to the survival group (*p* < 0.05). In contrast, variables such as sex, age, maximum SpO_2_, maximum RBC, mean platelet count, maximum HGB, minimum anion gap, maximum sodium, mean sodium, minimum glucose, maximum glucose, mean glucose, minimum potassium, mean chloride, maximum calcium, minimum creatinine, and mean temperature showed no statistically significant differences between the survival and non-survival groups (*p* > 0.05).

**Table 1:** Baseline characteristics, vital signs, laboratory parameters and statistic results of eICU patients.

Variable	Total (*n* = 5,383)	Survival (*n* = 5,097)	Death (*n* = 286)	*p*
Gender, *n* (%)				
0	2,559 (47.5)	2,423 (47.5)	136 (47.6)	1
1	2,824 (52.5)	2,674 (52.5)	150 (52.4)	
Sapsii score, median (Q1, Q3)	16.0 (12.0–18.0)	16.0 (12.0–18.0)	18.0 (15.0–22.0)	<0.001
Sofa score, median (Q1, Q3)	2.0 (1.0–3.0)	1.0 (0.0–3.0)	3.0 (1.0–5.0)	<0.001
Age, median (Q1, Q3)	71.0 (61.0–79.0)	71.0 (61.0–79.0)	74.0 (65.0–81.0)	0.15477
Min SpO_2_, median (Q1, Q3)	83.0 (74.0–89.0)	84.0 (75.0–89.0)	59.0 (38.8–73.0)	<0.001
Max SpO_2_, median (Q1, Q3)	100.0 (100.0–100.0)	100.0 (100.0–100.0)	100.0 (100.0–100.0)	1
Mean SpO_2_, median (Q1, Q3)	96.3 (94.9–97.7)	96.4 (95.0–97.7)	95.1 (92.7–96.7)	<0.001
Min WBC, median (Q1, Q3)	7.2 (5.6–9.3)	7.2 (5.6–9.2)	8.4 (6.3–10.8)	<0.001
Max WBC, median (Q1, Q3)	11.6 (8.6–15.4)	11.5 (8.5–15.1)	15.9 (11.7–21.9)	<0.001
Mean WBC, median (Q1, Q3)	9.3 (7.2–11.8)	9.2 (7.2–11.6)	12.2 (9.1–14.8)	<0.001
Min RBC, median (Q1, Q3)	3.5 (2.9–4.1)	3.5 (3.0–4.1)	3.2 (2.7–3.9)	<0.001
Max RBC, median (Q1, Q3)	4.0 (3.5–4.6)	4.1 (3.5–4.6)	3.9 (3.4–4.5)	0.34971
Mean RBC, median (Q1, Q3)	3.7 (3.2–4.3)	3.7 (3.2–4.3)	3.5 (3.0–4.0)	0.00317
Min platelets, median (Q1, Q3)	176.0 (132.0–226.2)	177.0 (133.0–227.0)	151.0 (103.0–208.0)	<0.001
Max platelets, median (Q1, Q3)	240.0 (186.0–307.0)	240.0 (186.0–306.0)	242.0 (184.0–316.0)	1
Mean platelets, median (Q1, Q3)	205.0 (159.0–258.7)	206.0 (159.6–259.2)	196.2 (149.2–247.5)	1
Min HGB, median (Q1, Q3)	10.0 (8.5–11.7)	10.0 (8.5–11.8)	9.2 (7.8–10.8)	<0.001
Max HGB, median (Q1, Q3)	11.7 (10.3–13.4)	11.8 (10.3–13.5)	11.3 (9.9–13.3)	1
Mean HGB, median (Q1, Q3)	10.7 (9.3–12.4)	10.7 (9.4–12.5)	10.1 (8.9–11.8)	0.0126
Min anion gap, median (Q1, Q3)	8.0 (6.0–11.0)	8.0 (5.9–11.0)	8.6 (6.0–12.0)	0.82852
Max anion gap, median (Q1, Q3)	13.0 (10.3–16.0)	13.0 (10.0–16.0)	16.0 (12.0–20.0)	<0.001
Mean anion gap, median (Q1, Q3)	10.4 (8.0–13.1)	10.3 (8.0–13.0)	12.5 (9.0–15.3)	<0.001
Min sodium, median (Q1, Q3)	135.0 (132.0–138.0)	135.0 (132.0–138.0)	134.0 (130.0–137.0)	0.00685
Max sodium, median (Q1, Q3)	141.0 (138.0–143.0)	141.0 (138.0–143.0)	141.0 (137.0–146.0)	1
Mean sodium, median (Q1, Q3)	138.0 (135.2–140.4)	138.0 (135.3–140.4)	137.4 (134.1–140.8)	1
Min glucose, median (Q1, Q3)	89.0 (74.0–105.0)	89.0 (75.0–105.0)	88.0 (68.0–111.2)	1
Max glucose, median (Q1, Q3)	211.0 (153.0–293.0)	210.0 (152.8–293.0)	214.0 (162.8–293.2)	1
Mean glucose, median (Q1, Q3)	136.4 (114.1–169.3)	136.0 (113.7–169.2)	141.1 (120.9–171.7)	1
Min potassium, median (Q1, Q3)	3.6 (3.2–3.9)	3.6 (3.2–3.9)	3.6 (3.2–4.2)	1
Max potassium, median (Q1, Q3)	4.7 (4.3–5.2)	4.7 (4.3–5.2)	5.1 (4.6–5.8)	<0.001
Mean potassium, median (Q1, Q3)	4.1 (3.8–4.4)	4.1 (3.8–4.4)	4.3 (3.9–4.8)	<0.001
Min chloride, median (Q1, Q3)	97.0 (93.0–101.0)	97.0 (93.0–101.0)	96.0 (92.0–100.0)	1
Max chloride, median (Q1, Q3)	104.0 (100.0–107.0)	103.0 (100.0–107.0)	104.0 (100.0–109.0)	1
Mean chloride, median (Q1, Q3)	100.3 (96.7–103.6)	100.2 (96.7–103.5)	100.4 (96.1–104.1)	1
Min bicarbonate, median (Q1, Q3)	24.0 (21.0–28.0)	24.0 (21.0–28.0)	21.0 (17.0–26.0)	<0.001
Max bicarbonate, median (Q1, Q3)	30.0 (27.0–34.3)	30.0 (27.0–35.0)	28.0 (25.0–33.0)	<0.001
Mean bicarbonate, median (Q1, Q3)	27.3 (24.4–30.8)	27.5 (24.6–31.0)	24.5 (22.0–29.4)	<0.001
Min calcium, median (Q1, Q3)	8.3 (7.9–8.7)	8.4 (7.9–8.7)	7.9 (7.3–8.5)	<0.001
Max calcium, median (Q1, Q3)	9.1 (8.7–9.5)	9.1 (8.8–9.5)	9.0 (8.5–9.5)	0.18182
Mean calcium, median (Q1, Q3)	8.7 (8.4–9.1)	8.7 (8.4–9.1)	8.5 (8.0–8.9)	<0.001
Min bun, median (Q1, Q3)	23.0 (16.0–36.0)	23.0 (16.0–35.0)	32.0 (20.0–44.2)	<0.001
Max bun, median (Q1, Q3)	42.0 (28.0–63.0)	41.0 (27.0–62.0)	62.0 (44.8–85.2)	<0.001
Mean bun, median (Q1, Q3)	33.0 (22.4–48.4)	32.3 (22.0–47.6)	45.6 (32.2–61.5)	<0.001
Min creatinine, median (Q1, Q3)	1.2 (0.9–1.8)	1.2 (0.8–1.8)	1.3 (0.9–1.9)	1
Max creatinine, median (Q1, Q3)	1.7 (1.2–2.8)	1.7 (1.2–2.8)	2.4 (1.7–3.5)	<0.001
Mean creatinine, median (Q1, Q3)	1.4 (1.0–2.2)	1.4 (1.0–2.2)	1.8 (1.3–2.6)	<0.001
Min albumin, median (Q1, Q3)	2.9 (2.5–3.4)	3.0 (2.5–3.4)	2.5 (2.1–2.9)	<0.001
Max albumin, median (Q1, Q3)	3.3 (2.9–3.7)	3.4 (3.0–3.7)	3.1 (2.6–3.5)	<0.001
Mean albumin, median (Q1, Q3)	3.1 (2.7–3.5)	3.1 (2.8–3.5)	2.8 (2.4–3.1)	<0.001
Min alt SGPT, median (Q1, Q3)	21.0 (14.0–34.0)	21.0 (14.0–34.0)	24.0 (15.0–53.0)	0.01158
Max alt SGPT, median (Q1, Q3)	27.0 (17.0–49.8)	26.0 (17.0–47.0)	52.0 (24.0–180.0)	<0.001
Mean alt SGPT, median (Q1, Q3)	24.0 (16.0–42.5)	24.0 (16.0–40.0)	41.9 (21.0–112.8)	<0.001
Min total bilirubin, median (Q1, Q3)	0.6 (0.4–0.9)	0.6 (0.4–0.9)	0.8 (0.5–1.2)	<0.001
Max total bilirubin, median (Q1, Q3)	0.7 (0.5–1.2)	0.7 (0.5–1.2)	1.2 (0.7–2.2)	<0.001
Mean total bilirubin, median (Q1, Q3)	0.6 (0.4–1.0)	0.6 (0.4–1.0)	1.0 (0.6–1.6)	<0.001
Min HR, median (Q1, Q3)	65.0 (56.0–74.0)	65.0 (57.0–74.0)	59.0 (30.5–72.5)	<0.001
Max HR, median (Q1, Q3)	107.0 (92.0–125.0)	106.0 (92.0–123.0)	128.0 (109.0–144.0)	<0.001
Mean HR, median (Q1, Q3)	83.0 (73.5–93.5)	82.7 (73.2–93.0)	90.7 (80.6–103.8)	<0.001
Min RR, median (Q1, Q3)	13.0 (10.0–16.0)	13.0 (10.0–16.0)	9.0 (0.0–14.0)	<0.001
Max RR, median (Q1, Q3)	31.0 (26.0–38.0)	30.0 (26.0–37.0)	36.0 (30.0–42.8)	<0.001
Mean RR, median (Q1, Q3)	20.2 (18.3–22.6)	20.1 (18.3–22.5)	21.3 (18.6–24.7)	<0.001
Min SBP, median (Q1, Q3)	90.0 (77.0–104.0)	90.0 (78.0–105.0)	68.0 (54.0–83.0)	<0.001
Max SBP, median (Q1, Q3)	158.0 (139.0–180.0)	158.0 (139.5–180.0)	148.0 (128.0–167.0)	<0.001
Mean SBP, median (Q1, Q3)	121.6 (108.1–136.8)	122.7 (109.1–137.6)	105.5 (95.2–117.7)	<0.001
Min DBP, median (Q1, Q3)	45.0 (36.0–54.0)	45.0 (36.0–54.0)	33.0 (22.0–46.0)	<0.001
Max DBP, median (Q1, Q3)	97.0 (84.0–112.0)	97.0 (84.0–112.0)	96.0 (81.0–113.0)	1
Mean DBP, median (Q1, Q3)	65.6 (58.7–73.4)	65.9 (59.0–73.8)	60.5 (54.1–66.0)	<0.001
Min temp, median (Q1, Q3)	36.1 (35.8–36.4)	36.2 (35.8–36.4)	36.0 (35.5–36.3)	<0.001
Max temp, median (Q1, Q3)	37.2 (37.0–37.7)	37.2 (37.0–37.7)	37.6 (37.0–38.4)	<0.001
Mean temp, median (Q1, Q3)	36.7 (36.5–36.9)	36.7 (36.5–36.9)	36.7 (36.4–37.1)	1

HGB, hemoglobin; ALT SGPT, alanine aminotransferase/serum glutamic - pyruvic transaminase.

We employed a feature selection strategy based on Boruta combined with feature importance. Traditional feature selection methods, such as Pearson correlation and stepwise selection, primarily capture linear relationships and often fail to account for interactions among variables (27–29). Therefore, we adopted the Boruta algorithm in conjunction with feature importance. Boruta is an automated feature selection algorithm based on random forests that uses statistical methods to identify features significantly associated with the target variable. Its core principle involves generating “shadow features” as noise benchmarks to distinguish truly important variables. This method offers several advantages, including automated thresholding to reduce manual intervention, retention of weak but stable features, robustness to multicollinearity, enhanced model interpretability, and applicability to high-dimensional heterogeneous data.

We selected the following features: minimum SpO_2_, minimum SBP, mean SBP, minimum RR, minimum HR, minimum albumin, minimum DBP, maximum BUN, mean BUN, maximum HR, mean SpO_2_, maximum creatinine, maximum WBC, mean WBC, and mean DBP.

### Shapley additive explanations (SHAP)

The SHAP (SHapley Additive exPlanations) algorithm was applied to interpret the MLP model. Among the 15 selected features, the top 10 in terms of importance were min SpO_2_, min albumin, mean wbc, mean SpO_2_, mean sbp, mean bun, min rr, mean dbp, min hr, and min sbp. A reduction in minimum oxygen saturation (min SpO_2_) was generally associated with increased mortality risk. Lower levels of minimum albumin (min albumin) were also linked to increased mortality risk. The clinical significance of the mean white blood cell count (mean wbc) is complex; both abnormally low and elevated levels may be indicative of adverse outcomes. A decrease in average oxygen saturation (mean SpO_2_) may reflect inadequate oxygenation, thereby increasing the risk of mortality. Lower mean systolic blood pressure (mean sbp) was found to be associated with higher mortality. Elevated blood urea nitrogen (mean bun), potentially indicating renal dysfunction, was also linked to increased mortality. A decreased minimum respiratory rate (min rr) was associated with increased risk of death. Lower mean diastolic blood pressure (mean dbp) was related to higher mortality risk. Severely reduced minimum heart rate (min hr) was also associated with elevated mortality risk. Finally, decreased minimum systolic blood pressure (min sbp) was strongly associated with elevated mortality risk.

## Discussion

Upon ICU admission, a total of 61 features were collected from patients. To improve prediction accuracy, the collected data were processed to include minimum, maximum, and mean values, accounting for dynamic changes in patients' conditions and the evolving nature of mortality prediction. Data from the two groups were processed separately to preserve the independence of the datasets. The MIMIC database was not involved in model development and was used solely as an external test set.

**Figure 2:**
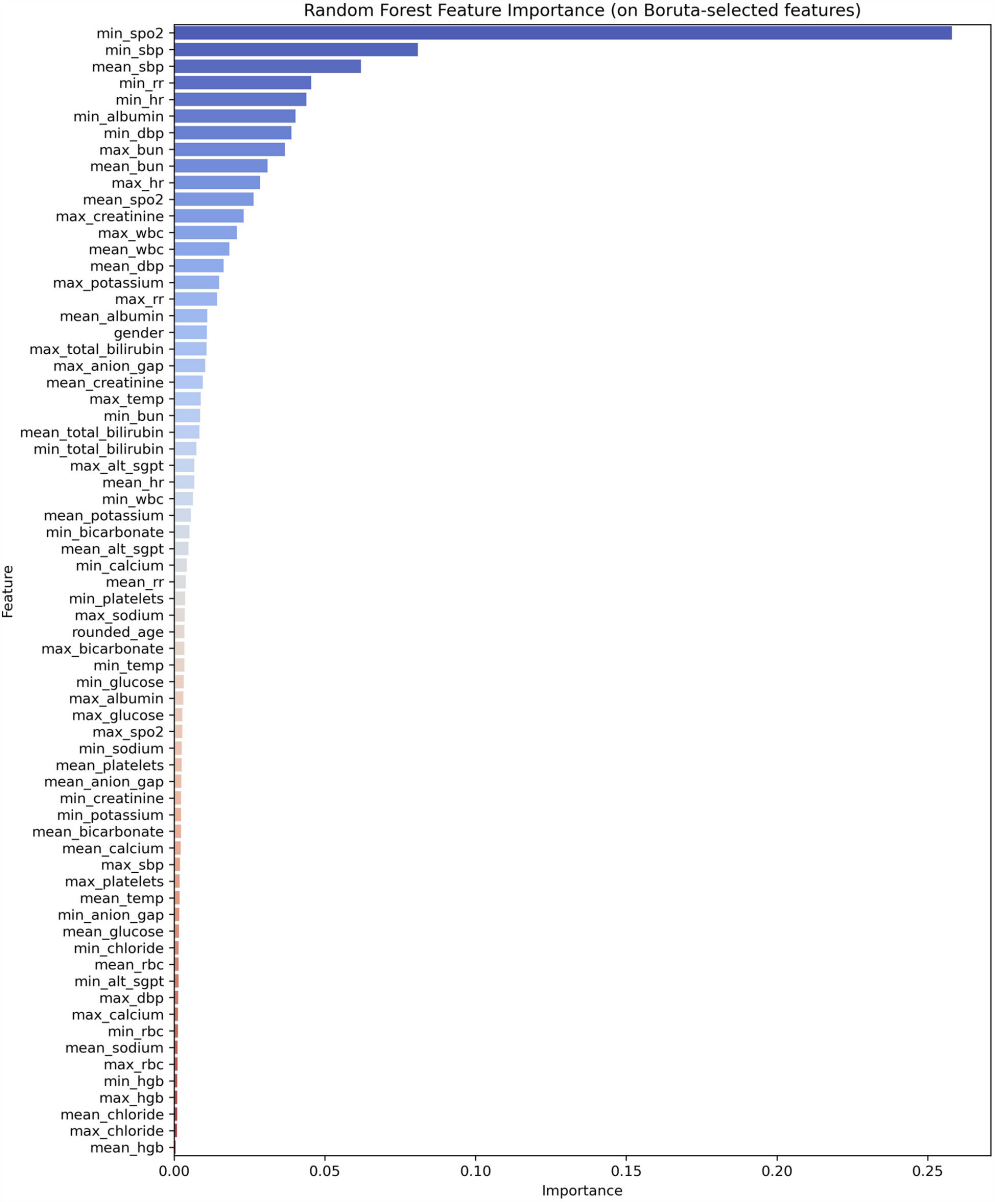
Feature importance ranking.

**Figure 3:**
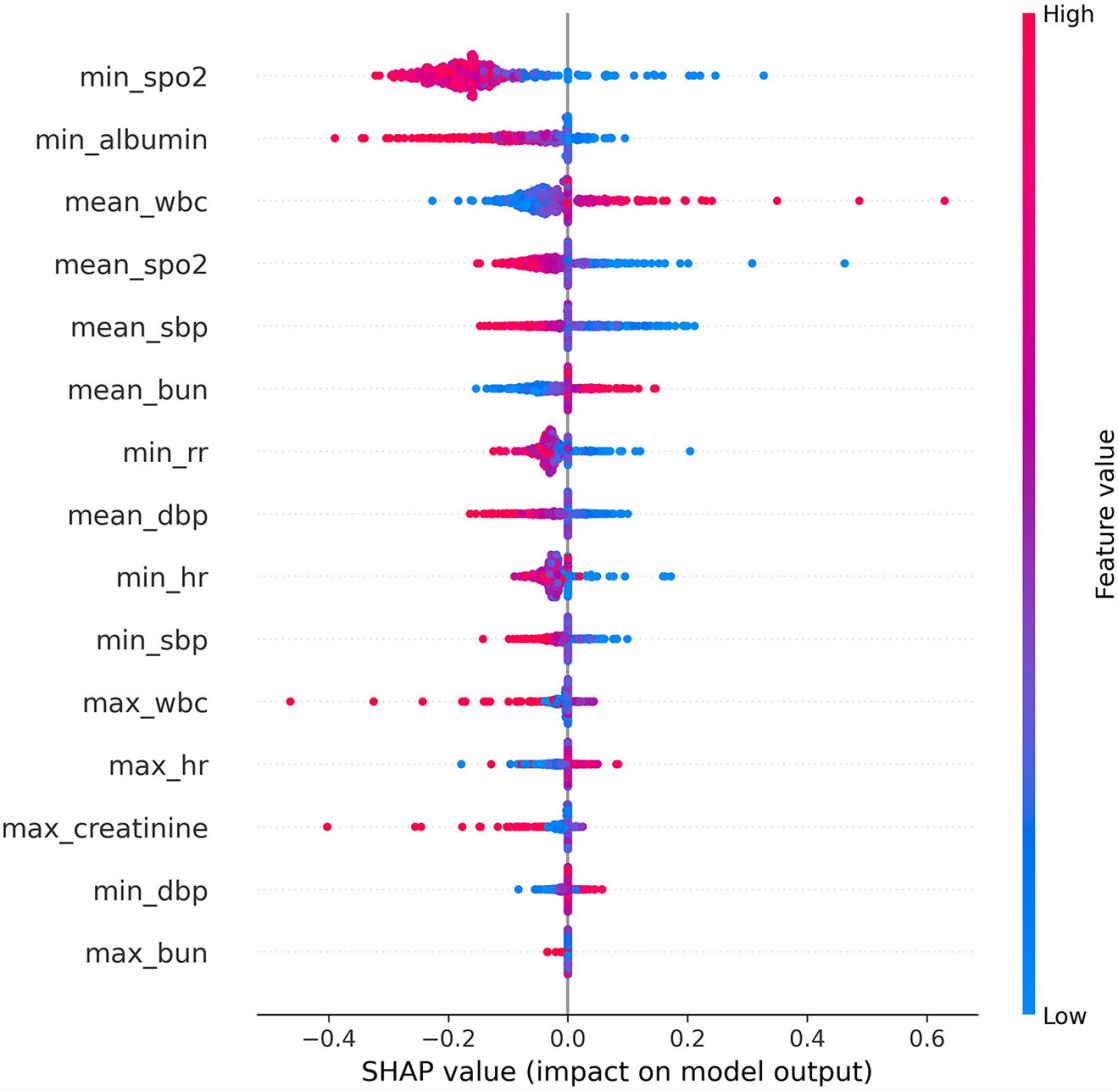
The shap analysis of MLP.

Through feature selection using Boruta and feature importance analysis, 15 features were identified Figure 2. Subsequently, the SHAP additive explanation method was applied to analyze the top-performing model (MLP) Figure 3. The top 10 features were identified as follows: minimum oxygen saturation (min SpO_2_), minimum albumin level (min albumin), mean white blood cell count (mean WBC), mean oxygen saturation (mean SpO_2_), mean systolic blood pressure (mean SBP), mean blood urea nitrogen (mean BUN), minimum respiratory rate (min RR), mean diastolic blood pressure (mean DBP), minimum heart rate (min HR), and minimum systolic blood pressure (min SBP).

**Figure 4:**
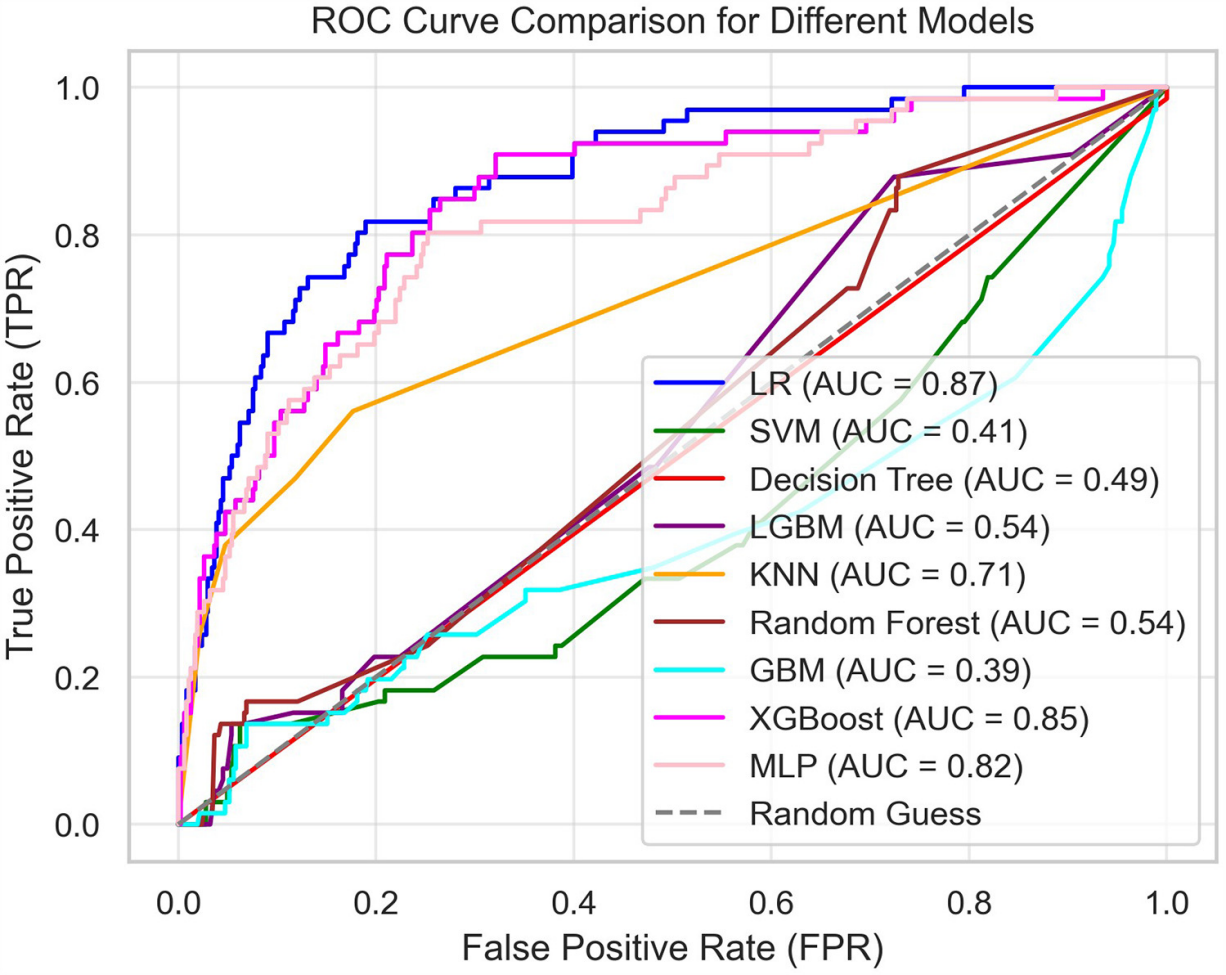
Summary of the ROC curves of all models.

**Figure 5:**
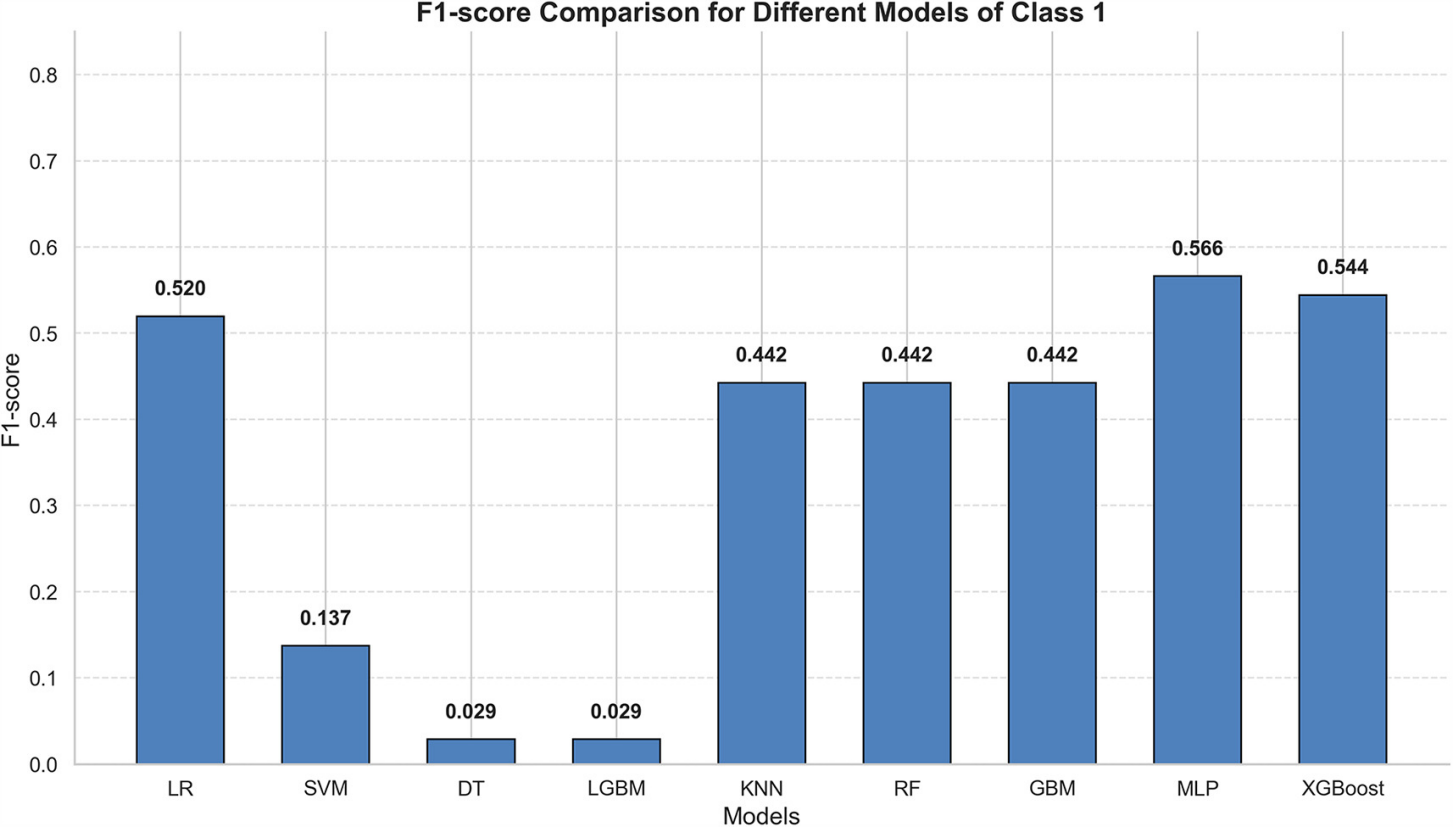
Summary of f1 scores of all models.

Heart failure patients exhibit significant clinical heterogeneity, and multiple treatment strategies are available. Rapid prediction of patient mortality would allow for timely adjustment of treatment plans, thereby significantly improving patient outcomes. In this study, nine models were selected: Logistic Regression, SVM, Decision Tree, LGBM, KNN, Random Forest, GBM, XGBoost, and MLP. These models were trained using random search and cross-validation. While AUC reflects the overall predictive accuracy, it is limited in assessing performance on the minority class (Figure 4). Therefore, the F1-score was selected as the primary evaluation metric, as it more accurately captures the model's ability to predict mortality by providing a better balance between false negatives (FN) and false positives (FP) (Figure 5) (30, 31).

In this study, the eICU dataset was used as the development set to train the models, while the MIMIC-IV dataset served as an independent external test set to evaluate model performance. Each dataset underwent preprocessing: eICU data were handled through missing value imputation, multiple imputation, oversampling, and data normalization. The models were then trained under cross-validation. Since tree-based models do not require data normalization, the Decision Tree, Light Gradient Boosting Machine (LGBM), Random Forest, Gradient Boosting Machine (GBM), and Extreme Gradient Boosting (XGBoost) were not normalized. After missing value handling, the MIMIC-IV dataset was also used with unnormalized data for prediction in the tree-based models. Ultimately, the Multilayer Perceptron (MLP) demonstrated the best predictive performance, providing evidence of its ability to predict patient mortality risk Table 2. Although machine learning models must still be integrated with clinical decision-making, they can offer valuable auxiliary support in the absence of detailed examinations.

**Table 2:** The final scores of all models.

Models	Accuracy	Precision (1)	Recall (1)	F1 (1)	Macro F1	Weighted F1
LR	0.88	0.54	0.50	0.52	0.73	0.88
SVM	0.81	0.18	0.14	0.15	0.52	0.80
DT	0.87	0.47	0.32	0.38	0.65	0.86
LGBM	**0** **.** **89**	**0.75**	0.14	0.23	0.58	0.85
KNN	0.88	0.53	0.38	0.44	0.69	0.87
RF	0.88	0.62	0.20	0.30	0.62	0.86
GBM	0.88	0.67	0.12	0.21	0.57	0.85
MLP	0.87	0.49	**0** **.** **64**	**0** **.** **56**	**0** **.** **74**	**0** **.** **88**
XGBoost	0.86	0.47	**0** **.** **65**	0.54	0.73	0.87

Bold font indicates the highest value achieved for this metric.

A decrease in minimum saturation of peripheral oxygen (min SpO_2_) is associated with an increased risk of mortality, as low oxygen saturation reflects impaired cardiopulmonary function, which affects oxygen supply and poses a life-threatening risk (32, 33). A reduction in minimum albumin (min albumin) may also increase the risk of mortality, as low albumin levels suggest poor nutritional status or impaired liver synthesis, indicating overall body dysfunction (34, 35). The significance of mean white blood cell count (mean wbc) is more complex; a low count may increase mortality risk due to immune system dysfunction, while a high count may indicate an excessive inflammatory response or other adverse factors, which does not necessarily correlate with a reduction in risk (36, 37). A decrease in mean saturation of peripheral oxygen (mean SpO_2_) is linked to an increased risk of mortality, as adequate oxygenation is essential for vital functions, and a low mean oxygen saturation suggests inadequate oxygen supply (38). A reduction in mean systolic blood pressure (mean sbp) may also increase mortality risk, as low systolic blood pressure reflects issues with cardiac output or peripheral circulation (39, 40). An elevated mean blood urea nitrogen (mean bun) level may indicate impaired renal function, thus increasing mortality risk; however, a lower value is generally considered favorable, though it is not necessarily inversely correlated with mortality (40). A significantly low minimum respiratory rate (min rr) may indicate severe suppression of respiratory function, increasing mortality risk (41, 42). A decrease in mean diastolic blood pressure (mean dbp) may reflect cardiovascular abnormalities and is associated with an increased risk of mortality (43). A very low minimum heart rate (min hr) may indicate severe cardiac dysfunction, leading to a higher mortality risk (44, 45). A decrease in minimum systolic blood pressure (min sbp) typically reflects a sudden deterioration in cardiac pumping ability or peripheral circulation, thus increasing the risk of mortality.

The study has several limitations. First, this is a retrospective study, which is subject to selection bias and has not been validated by prospective research. Second, the model used in this study was derived from existing databases and is therefore limited by the quality of the original data. Third, the F1-score of the study remains low, which limits its clinical applicability.

## Conclusion

Among the nine models, the Multi-Layer Perceptron (MLP) achieved the highest recall rate (0.64), identifying the greatest number of true positives and minimizing the risk of missed diagnoses. It also exhibited superior overall performance, with the highest Macro F1 score (0.74) and Weighted F1 score (0.88), effectively balancing performance across all categories.

## Data Availability

Publicly available datasets were analyzed in this study. This data can be found here: https://physionet.org/content/mimiciii/1.4, https://physionet.org/content/eicu-crd/2.0/.
